# Data of 10 SSR markers for genomes of homo sapiens and monkeys

**DOI:** 10.1016/j.dib.2017.04.010

**Published:** 2017-04-13

**Authors:** K.K.V.V.V.S. Reddy, S.Viswanadha Raju, Chinta Someswara Rao

**Affiliations:** a Rayalasheema University, AP, India; b Department of CSE, JNTUHCEJ, JNT University Hyderabad, Telangana, India; c Department of CSE, SRKR Engineering College, Bhimavaram, AP, India

**Keywords:** SSR, Genomes, Homo sapiens, Monkeys

## Abstract

In this data, we present 10 Simple Sequence Repeat(SSR) markers TAGA, TCAT, GAAT, AGAT, AGAA, GATA, TATC, CTTT, TCTG and TCTA which are extracted from the genomes of homo sapiens and monkeys using string matching mechanism [1]. All loci showed 4 Base Pair(bp) in allele size, indicating that there are some polymorphisms between individuals correlating to the number of SSR repeats that maybe useful for the detection of similarity among the genotypes. Collectively, these data show that the SSR extraction is a valuable method to illustrate genetic variation of genomes.

**Specifications Table**

**Table:** 

Subject area	Bio-informatics
More specific subject area	Genomes of homo sapiens and monkeys
Type of data	Tables, figures
How data was acquired	SSR markers extraction with string matching
Data format	Analyzed
Experimental factors	Ten SSR motifs: *TAGA, TCAT, GAAT, AGAT, AGAA, GATA, TATC, CTTT, TCTG, TCTA* were targeted. String matching process is applied on genomes of homo sapiens and monkeys. 10 SSR markers to be used in various detection purposes are extracted with this approach.
Experimental features	Each of the 10 SSR markers are extracted from genomes of homo sapiens and monkeys. All the 10 SSRs showed the 4 bp in allele size. These differences showed that there are some polymorphisms among the genomes to the number of SSR repeats.
Data source location	BHIMAVARAM, INDIA
Data accessibility	The data is provided with this article

**Value of the data**

• Data sets obtained from genomes of homo sapiens and monkeys with string matching have shown the high specificity.
• These data suggest that SSR extraction is an useful method for providing information for various detections.
• Access to the raw sequencing data allows researchers to perform further bio-informatics analysis based on their own computational algorithms.

## 1 Data

10 SSR markers data which were extracted from genomes of Homo sapiens and Monkeys are shown in Table 1. The data presented here shows that the SSR extraction with string matching was very useful and was able to reveal variation of selected genome collections. These SSR markers can be used to assess maternity, paternity, personal and theft identifications. All chromosomes of *Homo sapiens and monkeys (Callithrix jacchus, Chlorocebus sabaeus, Gorilla gorilla,Macaca fascicularis, Macacamulatta, Nomascus leucogenys, Pan troglodytes, Papio anubis and Pongo abelli)* are considered for the extraction of the 10 SSR markers which are shown in Table 1 [1].

### 1.1 10 SSR markers overall count in homo sapiens and monkeys

Table 2 shows the 10 SSRs overall count of *Callithrix jacchus, Chlorocebus sabaeus, Gorilla gorilla, Homo sapiens, Macaca fascicularis, Macacamulatta, Nomascus leucogenys, Pan troglodytes, Papio anubis and Pongo abelli respectively.*

**Fig. A1:**
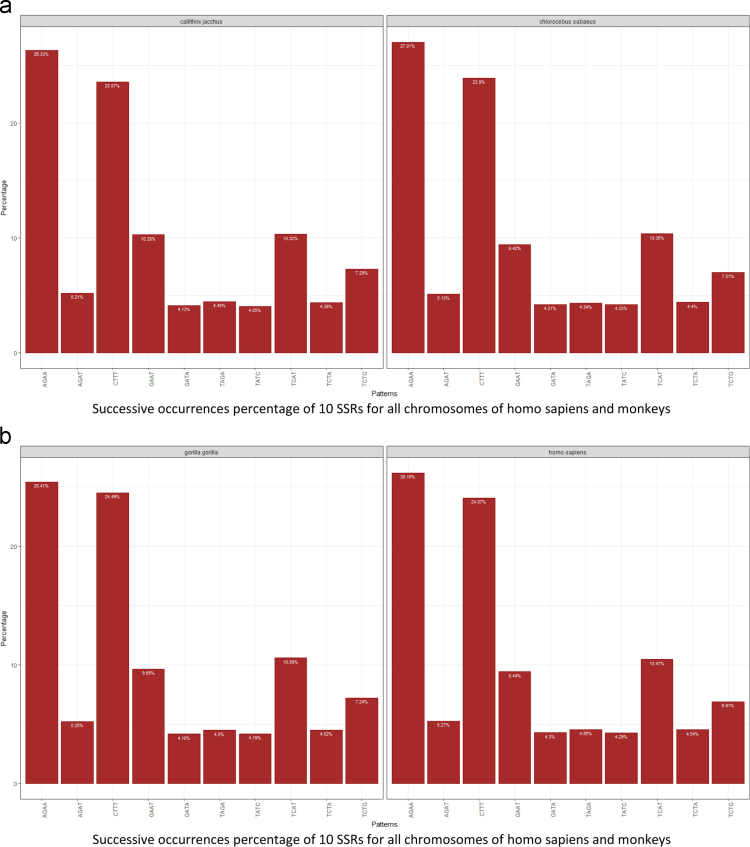

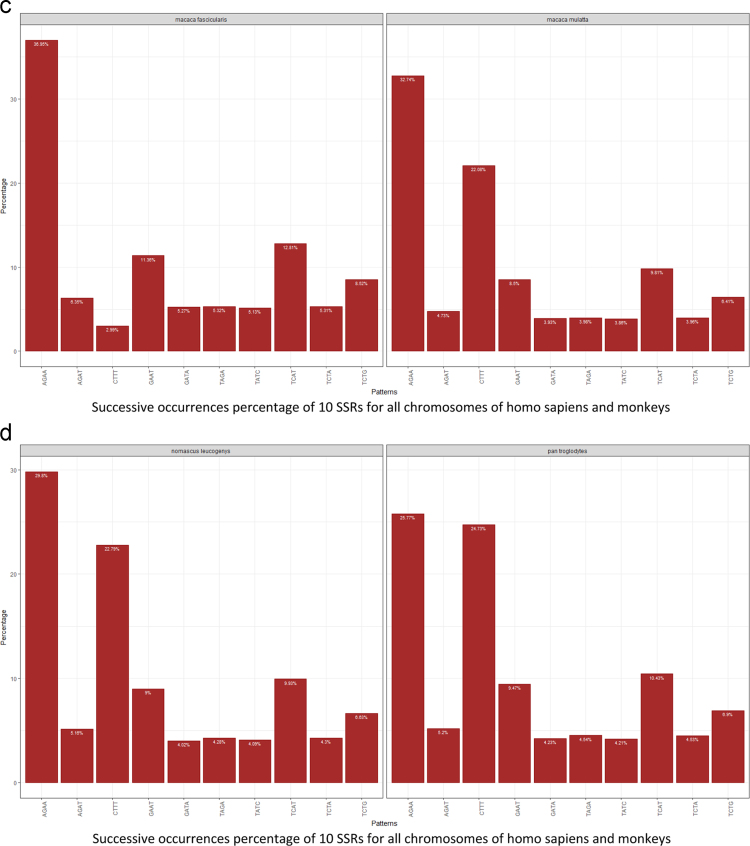

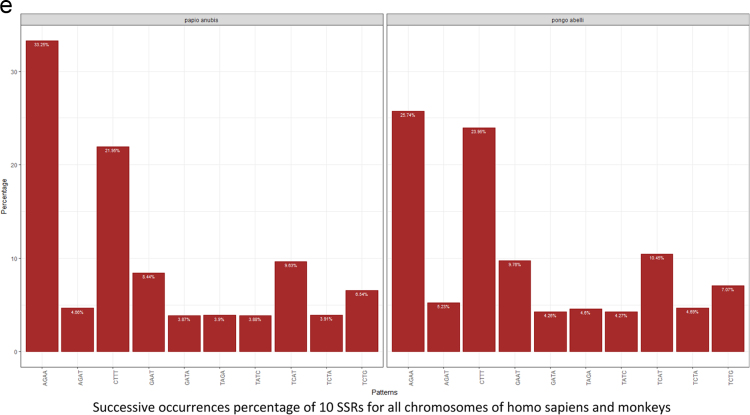
(a). Successive occurrences percentage of 10 SSRs for all chromosomes of homo sapiens and monkeys (b). Successive occurrences percentage of 10 SSRs for all chromosomes of homo sapiens and monkeys (c). Successive occurrences percentage of 10 SSRs for all chromosomes of homo sapiens and monkeys (d). Successive occurrences percentage of 10 SSRs for all chromosomes of homo sapiens and monkeys (e). Successive occurrences percentage of 10 SSRs for all chromosomes of homo sapiens and monkeys.

Fig. A1 (presented in Appendix A (figures part) from A1(a) to (e)) shows the successive occurrence percentage of 10 SSRs for all chromosomes of *Callithrix jacchus, Chlorocebus sabaeus, Gorilla gorilla, Homo sapiens, Macaca fascicularis, Macacamulatta, Nomascus leucogenys, Pan troglodytes, Papio anubis and Pongo abelli***.**

### 1.2 Position and MAX number of occurrences of 10 SSRs for each chromosome of homo sapiens and monkeys

Table A1 shows ( presented in Appendix A (tables part)) the 10 SSRs position and MAX number of occurrences for each chromosome of *Callithrix jacchus, Chlorocebus sabaeus, Gorilla gorilla, Homo sapiens, Macaca fascicularis, Macacamulatta, Nomascus leucogenys, Pan troglodytes, Papio anubis and Pongo abelli respectively.*

### 1.3 Data for all the chromosomes of homo sapiens and monkeys for every 10 SSR count

Table A2, Table A3, Table A4, Table A5, Table A6, Table A7, Table A8, Table A9, Table A10, Table A11 show (presented in Appendix A (tables part)) every SSR count for all chromosome of *Callithrix jacchus, Chlorocebus sabaeus, Gorilla gorilla, Homo sapiens, Macaca fascicularis, Macacamulatta, Nomascus leucogenys, Pan troglodytes, Papio anubis and Pongo abelli respectively*.

**Fig. A2:**
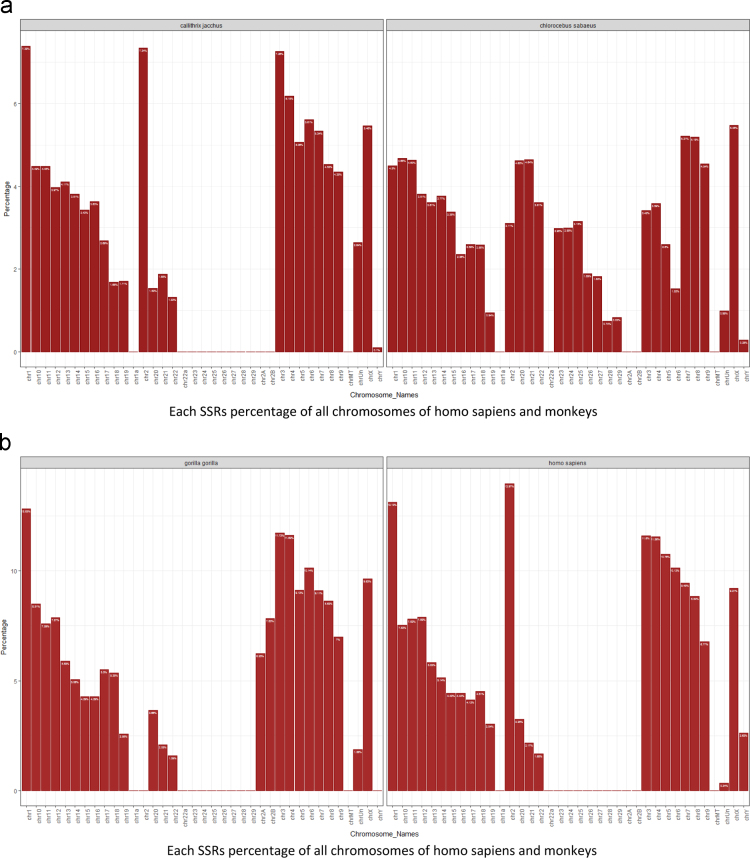

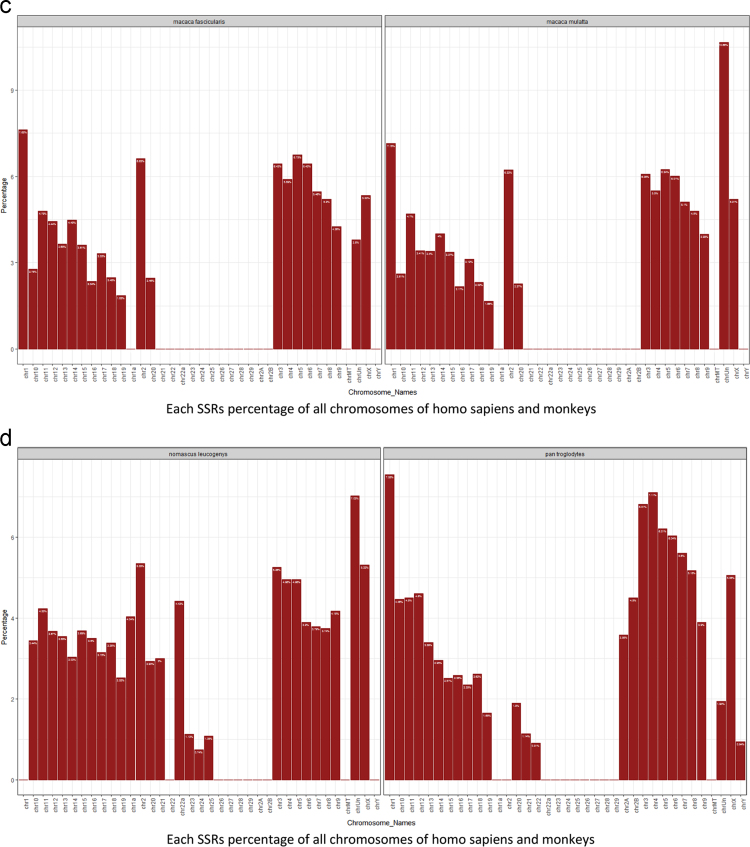

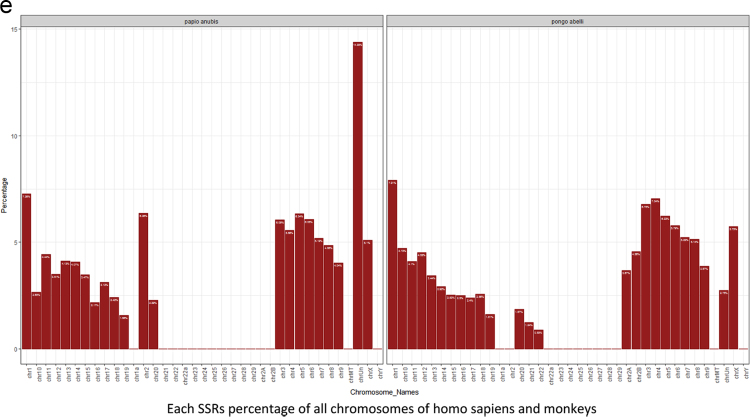
(a). Each SSRs percentage of all chromosomes of homo sapiens and monkeys (b). Each SSRs percentage of all chromosomes of homo sapiens and monkeys (c). Each SSRs percentage of all chromosomes of homo sapiens and monkeys (d). Each SSRs percentage of all chromosomes of homo sapiens and monkeys (e). Each SSRs percentage of all chromosomes of homo sapiens and monkeys.

Fig. A2 shows (presented in Appendix A (figure part) from A1(a) to A2(e)) the each SSRs percentage of all chromosomes of *Callithrix jacchus, Chlorocebus sabaeus, Gorilla gorilla, Homo sapiens, Macaca fascicularis, Macacamulatta, Nomascus leucogenys, Pan troglodytes, Papio anubis and Pongo abelli*.

## 2 Experimental design, materials and methods

### 2.1 SSR extraction

In this paper all chromosomes of *homo sapiens and monkeys(Callithrix jacchus, Chlorocebus sabaeus, Gorilla gorilla,Macaca fascicularis, Macacamulatta, Nomascus leucogenys, Pan troglodytes, Papio anubis and Pongo abelli*) and the ten(*TAGA, AGAA, GATA, TCTA, TCAT, GAAT, AGAT, CTTT, TATC, TCTG*) SSRs are considered. SSRs are extracted from homo sapiens and monkeys using string matching approach. The string matching is a searching mechanism that searches the repeats in a given chromosomal file.

*Search process:* The chromosomes and SSRs are given to main function, then the main function calls the shift process by providing right most character of the SSRs. The shift position is returned to main function by the shift process. The search process compares character by character from both the directions until a complete match or mismatch occurs. If match occurs the successive occurrence of the pattern is searched. If the successive occurrence size is greater than 1 then the data is stored in database [1]. This process is continued for all the SSRs and for entire data in the chromosomes. The detailed description is given in [1].

### 2.2 Paternity identification with similarity measures [2]

In cases related to paternity tests, two or more persons might claim that a child is their biological son/daughter. In such cases, the genome sequence of the child as well as the persons can be compared to identify the similarity of the loci that is stored in the Tandem Repeat Database(TandemRepeatDB). The person having more similarity of the loci with the child DNA will be considered to be the actual biological father/mother.

***Procedure:***

• Genome sequence of the child as well as the persons(A and B) is taken.
• The continuously occurred 10 loci׳s from child and persons (A and B) are extracted and stored in *TandemRepeatDB* using *multiple pattern multiple(2*^*N*^*) shaft parallel string matching algorithms* [3].
• The loci from *TandemRepeatDB* are extracted.
• Correlation coefficient, Rank correlation coefficient and Cosine similarity measures are applied to measure the similarity between loci of child and persons(A and B).
• Similarity measures return the percentage of similarity between the loci of child and persons (A and B).
• Using the similarity percentage, the similarity can be noticed in both the positive and negative terms.

Example of similarity between child and persons (A and B) is shown in Table 3.

In Table 3, correlation coefficient, rank correlation coefficient and cosine similarity measures show a positive correlation (1) between the child and person A, whereas between the child and person B show a positive correlation for all the three measures but it is very low compared to child and person A.

### 2.3 DNA finger printing

Performing pattern search in the entire genome of an organism in traditional approach i.e., using laboratory experiments is very time consuming. Even for a small part of a genome, the process will take several hours, moreover the related laboratory experiments are quite expensive. Due to the latest developments in genome sequencing, in the near future, a person can get their entire genome sequenced in a diagnostics centre just like the medical diagnostics. In this situation, the *multiple pattern multiple(2*^*N*^*) shaft parallel string matching algorithms* [3] will play a key role to search the loci in the person׳s genome and will return the occurrence positions, chromosome name, loci name etc., in a quick time and at no cost.

*DNA finger printing*—It is a method used to identify an individual from sample genome sequence by searching the patterns in the locations on all chromosomes.

***DNA finger printing procedure:***

• Genome sequences of one family members (father, mother, daughters and sons) are considered.
• The 10 loci in all the family members genomes are searched using *multiple pattern multiple(2*^*N*^*) shaft parallel string matching algorithms* [3].
• If exact match occurs then successive logic is applied.
• If successive occurrence of the loci is found then its sample name, position, chromosome name, pattern and number of times of occurrence related to all family members genomes are stored in TandemRepeatDB [1]
• The loci of all family members are extracted from *TandemRepeatDB*, their position, chromosome name, pattern and number of times of occurrence is compared.
• If they are matched then where the genomes of father and mother are matched to their child are shown.

## Figures and Tables

**Table A1:** The 10 SSRs position and MAX number of occurrences for each chromosome of homo sapiens and monkeys.

	**callithrix_jacchus**			**chlorocebus_sabaeus**			**gorilla_gorilla**		
**SSRs**	**Chr.**	**Position**	**MAX**	**Chr.**	**Position**	**MAX**	**Chr.**	**Position**	**MAX**
	**Name**		**No.occ.**	**Name**		**No.occ.**	**Name**		**No.occ.**
**TAGA**	chr3	79735472	21	chr21	6186496	19	chr15	38646887	19
				chr7	104177388	19	chr3	191310396	19
							chr6	67454746	19
**TCAT**	chr4	2828416	14	chr22	79937353	14	chr14	42977818	14
**GAAT**	chr16	10668381	13	chr6	37074500	14	chr1	19404908	12
							chr1	38217247	12
							chr1	52534114	12
							chr10	118648631	12
							chr11	21903713	12
							chr4	92024092	12
							chr6	41503923	12
							chr6	71087014	12
							chr6	168203280	12
							chr8	132267085	12
**AGAT**	chr3	79735473	21	chr21	6186493	20	chr15	38646884	20
							chr6	67454743	20
**AGAA**	chr18	5525740	57	chr14	8606607	54	chr3	164366107	41
**GATA**	chr3	79735474	20	chr21	6186494	20	chr15	38646885	20
**TATC**	chr1	193890637	18	chr20	32463615	20	chr10	93616477	26
	chr10	89477523	18						
	chrX	35314334	18						
**CTTT**	chr7	13494352	51	chr13	12914036	42	chrUn	33064271	66
**TCTG**	chr12	116706355	14	chr6	39751979	14	chr12	65943038	16
**TCTA**	chr10	89477521	18	chr11	17593555	20	chr10	93616475	26
	chrX	35314332	18	chr20	32463617	20			
				chr13	12914036	42			
	**homo_sapiens**			**macaca_fascicularis**			**macaca_mulatta**		
**TAGA**	chr6	78744943	21	chr8	68082659	29	chr1	102404825	31
**TCAT**	chr18	19040969	12	chr3	70869451	19	chr11	121369418	19
	chr19	5705151	12						
**GAAT**	chr7	24447326	12	chr12	9903931	14	chr1	207627818	15
				chr9	130094610	14			
				chrX	125482762	14			
**AGAT**	chr6	78744944	21	chr8	68082660	28	chr1	102404826	31
**AGAA**	chr1	59152850	42	chr6	30665766	218	chr1	208426961	84
	chr2	202039752	42						
**GATA**	chr6	78744941	22	chr8	68082657	29	chr1	102404827	31
**TATC**	chrX	2745391	25	chr2	190939258	33	chr2	145642434	21
**CTTT**	chr1	183001776	78	chr7	135702116	221	chr17	31653528	79
**TCTG**	chr16	6958183	12	chr1	183808952	16	chr15	16587493	12
	chr3	128159380	12				chr8	106806405	12
	chr6	42209966	12						
	chrX	33066905	12						
**TCTA**	chrX	2745393	25	chr2	190939260	33	chr11	139206709	21
							chr2	145642432	21
	**nomascus_leucogenys**			**pan_troglodytes**			**papio_anubis**		
**TAGA**	chr17	99694422	17	chr22	18705754	17	chr15	67841064	31
	chr20	37429530	17	chr4	114121219	17			
	chrX	134452351	17	chr7	30208909	17			
				chr8	117565722	17			
**TCAT**	chr11	76660754	12	chr11	66703196	10	chr3	35336353	15
				chr5	81454376	10			
				chr5	98961452	10			
				chr5	146242524	10			
				chr9	103588094	10			
**GAAT**	chr8	107360456	11	chr3	49868231	11	chr20	61370806	14
**AGAT**	chr20	37429531	17	chr7	30208906	18	chr15	67841061	32
**AGAA**	chr11	110907513	52	chr12	112420396	43	chr16	45124738	54
**GATA**	chr20	37429532	17	chr7	30208907	18	chr15	67841062	31
				chr8	117565720	18			
**TATC**	chr18	90384956	23	chr3	85098744	19	chr1	42020452	21
**CTTT**	chr11	71987795	33	chr11	122385983	30	chr19	41169691	47
**TCTG**	Chr22a	98163100	13	chr22	6919111	13	chr1	145463529	15
**TCTA**	chr18	90384958	22	chr3	85098746	18	chr2	91114291	22
	**pongo_abelli**								
**TAGA**	chr10	51304347	20						
**TCAT**	chr20	37504198	12						
**GAAT**	chr14	51525042	13						
**AGAT**	chr10	51304348	20						
**AGAA**	chr9	86034594	37						
**GATA**	chr10	51304349	19						
**TATC**	chr4	171960662	19						
**CTTT**	chr16	13498905	63						
**TCTG**	chr2B	108490608	11						
	chr3	129689645	11						
**TCTA**	chr2A	47115325	18						
	chr4	171960664	18						

**Table A2:** The 10 SSR counts for all the chromosomes of callithrix_jacchus.

	**TAGA**	**TCAT**	**GAAT**	**AGAT**	**AGAA**	**GATA**	**TATC**	**CTTT**	**TCTG**	**TCTA**
**chr1**	4120	9491	9433	4815	23363	3818	3754	20746	6704	4079
**chr2**	4028	9127	9149	4663	24108	3678	3693	20964	6155	4071
**chr3**	4363	9031	9048	4821	23465	3975	3798	20736	5322	4158
**chr4**	3394	7768	7769	3764	20392	3012	3048	17926	5143	3306
**chr5**	2469	6441	6405	2908	16343	2227	2156	15126	5301	2409
**chr6**	2952	7086	6983	3524	18332	2808	2802	16372	4644	2985
**chr7**	2838	7093	7017	3365	16919	2666	2396	15474	4874	2553
**chr8**	2475	5641	5654	3024	14685	2426	2310	12851	3785	2444
**chr9**	2306	5439	5431	2797	13781	2215	2233	12650	3952	2381
**chr10**	2396	5715	5668	2803	14578	2109	2138	12965	4198	2276
**chr11**	2355	5836	5888	2848	14173	2160	2199	12457	4378	2376
**chr12**	1992	5126	5036	2343	12582	1854	1786	11634	4184	1904
**chr13**	2223	5165	4983	2660	13253	2116	2054	12027	3514	2235
**chr14**	2010	4807	4598	2403	12301	1843	1828	11178	3408	2104
**chr15**	1768	4616	4399	2046	11082	1597	1565	9921	3129	1777
**chr16**	2059	4688	4711	2402	11486	1952	2002	10173	2850	2075
**chr17**	1427	3453	3483	1729	8742	1374	1301	7693	2212	1469
**chr18**	893	2115	2086	1019	5632	829	819	4800	1459	851
**chr19**	857	2232	2169	992	5539	798	748	5124	1640	819
**chr20**	905	1967	2020	939	4638	730	637	4499	1661	691
**chr21**	1137	2280	2135	1291	6081	1049	1077	5285	1466	1211
**chr22**	588	1481	1451	685	4427	516	540	4156	1739	587
**chrUn**	1249	2896	3208	1688	8391	1112	1258	8088	2890	1441
**chrX**	3489	6442	6724	4021	16932	3492	3282	14723	4261	3303
**chrY**	74	110	123	89	316	80	33	288	119	38

**Table A3:** The 10 SSR counts for all the chromosomes of chlorocebus_sabaeus.

	**TAGA**	**TCAT**	**GAAT**	**AGAT**	**AGAA**	**GATA**	**TATC**	**CTTT**	**TCTG**	**TCTA**
**chr1**	2388	6038	5637	2858	14992	2476	2281	13005	4118	2353
**chr2**	1585	4194	3852	1882	10256	1513	1538	9278	3129	1602
**chr3**	2080	4689	4053	2305	11168	1920	1985	9711	2692	2048
**chr4**	2099	4293	3853	2392	13253	1875	1938	10432	2667	2029
**chr5**	1202	3341	3125	1419	9061	1148	1109	8132	2644	1223
**chr6**	724	1759	1527	813	5192	658	663	5090	1856	686
**chr7**	3073	6687	6048	3644	17595	3013	2969	15238	3645	3119
**chr8**	2893	6815	6134	3528	17227	3036	2686	15312	4313	2797
**chr9**	2278	5831	5284	2726	15660	2181	2188	14041	4234	2309
**chr10**	2637	6137	5500	3087	15639	2434	2472	13986	3849	2617
**chr11**	2464	6096	5790	2989	15189	2401	2372	13848	4074	2573
**chr12**	2094	4979	4507	2491	12692	2021	1967	11296	3569	1964
**chr13**	2046	4727	4241	2291	12300	1859	1998	10776	2793	2035
**chr14**	1941	4941	4420	2317	12821	1966	1818	11233	3464	2080
**chr15**	1809	4455	3975	2224	11229	1800	1778	10315	2706	1875
**chr16**	1070	2782	2672	1286	8267	1005	1010	7561	2630	1117
**chr17**	1448	3279	2975	1646	8568	1361	1341	8082	2178	1466
**chr18**	1415	3424	3117	1627	8611	1393	1385	7549	2195	1442
**chr19**	427	1293	1195	511	3062	386	354	2907	1248	370
**chr20**	2302	6231	5876	2926	15377	2236	2156	13802	4564	2256
**chr21**	2560	5998	5495	3119	15798	2437	2453	13706	3769	2630
**chr22**	1958	4754	4522	2318	11758	1857	1922	10759	3159	2035
**chr23**	1591	4130	3641	1859	9754	1553	1515	8865	2629	1617
**chr24**	1662	3975	3616	1968	9626	1515	1467	9175	2717	1567
**chr25**	1664	4086	3766	1906	10752	1577	1701	9469	2708	1751
**chr26**	974	2491	2280	1127	6141	910	911	5779	1921	1009
**chr27**	1085	2421	2252	1256	6014	1029	1006	5169	1439	999
**chr28**	384	807	703	449	2604	370	440	2276	737	415
**chr29**	437	1043	885	506	2987	424	446	2410	759	435
**chrMT**		1			1		1			1
**chrUn**	477	548	419	534	4762	688	1164	2534	493	695
**chrX**	3287	6802	6007	3851	17890	3349	3581	15800	4278	3568
**chrY**	178	301	213	221	926	193	182	778	280	179

**Table A4:** The 10 SSR counts for all the chromosomes of gorilla_gorilla.

	**TAGA**	**TCAT**	**GAAT**	**AGAT**	**AGAA**	**GATA**	**TATC**	**CTTT**	**TCTG**	**TCTA**
**chr1**	4108	10443	9603	4753	23769	3742	3842	21773	6957	3964
**chr2A**	2055	4804	4254	2322	11643	1798	1868	10967	3289	1945
**chr2B**	2634	6026	5603	3051	14902	2402	2348	13264	3844	2504
**chr3**	3877	9278	8506	4334	21974	3577	3657	19894	5805	3895
**chr4**	4171	9255	8315	4846	21606	3974	3951	19205	4955	4106
**chr5**	2874	7092	6414	3289	17005	2581	2701	15865	5038	2857
**chr6**	3363	7748	7087	3975	19327	3124	3072	17172	4867	3374
**chr7**	2928	6835	6231	3377	16910	2739	2734	16419	4404	2857
**chr8**	2820	6767	6128	3411	16193	2813	2626	14573	4179	2805
**chr9**	2150	5177	4864	2427	12210	1910	1945	13716	3639	2088
**chr10**	2409	5988	5448	2676	14344	2217	2115	17363	4769	2987
**chr11**	2385	6066	5625	2856	13794	2248	2319	12882	4268	2488
**chr12**	2471	6055	5671	2981	14649	2408	2325	13783	3930	2501
**chr13**	2174	4798	4116	2413	10861	1971	1962	9747	2688	2087
**chr14**	1597	4042	3637	1933	9116	1549	1522	8931	2716	1538
**chr15**	1360	3429	3036	1508	8006	1215	1242	7328	2409	1366
**chr16**	1190	3446	3101	1404	7580	1127	1157	7755	2798	1282
**chr17**	1869	4008	3720	2127	10480	1754	1716	9335	2653	1877
**chr18**	1674	3427	3165	1802	8645	1471	1463	12163	2401	1571
**chr19**	599	1752	1513	735	4961	589	585	4823	1836	685
**chr20**	955	2729	2498	1086	5935	855	909	8050	2116	924
**chr21**	802	1528	1346	882	3918	705	662	3339	1067	738
**chr22**	403	1335	1286	483	2940	404	389	2566	1281	401
**chrMT**		3						1		2
**chrUn**	778	787	840	1604	2542	528	534	2704	2297	791
**chrX**	3386	6761	6088	3948	17643	3470	3582	16119	4372	3644

**Table A5:** The 10 SSR counts for all the chromosomes of homo_sapiens.

	**TAGA**	**TCAT**	**GAAT**	**AGAT**	**AGAA**	**GATA**	**TATC**	**CTTT**	**TCTG**	**TCTA**
**chr1**	4385	10928	10062	5083	25987	4016	4104	23464	7167	4258
**chr2**	4898	11132	9911	5595	27772	4439	4465	25243	7334	4596
**chr3**	4073	9446	8601	4549	23020	3773	3731	20670	5858	3995
**chr4**	4299	9393	8513	4946	22740	4138	4118	20193	5059	4316
**chr5**	3864	8541	7658	4449	21362	3557	3558	19007	5358	3914
**chr6**	3447	7963	7123	4021	20548	3254	3207	18297	4927	3462
**chr7**	3076	7173	6480	3596	18774	2904	2979	18061	4609	3115
**chr8**	3072	6837	6262	3610	17958	2991	2784	15811	4241	2990
**chr9**	2294	5498	5063	2583	13365	2034	2179	12119	3710	2279
**chr10**	2442	6037	5365	2810	14966	2321	2216	13720	4268	2473
**chr11**	2471	6290	5812	3021	15318	2449	2452	13998	4455	2665
**chr12**	2603	6126	5751	3079	15686	2512	2462	14606	3975	2610
**chr13**	2214	4820	4182	2491	11360	2039	2071	10206	2680	2181
**chr14**	1708	4202	3740	2064	9834	1618	1644	9566	2752	1706
**chr15**	1498	3618	3196	1676	8709	1355	1315	8057	2515	1480
**chr16**	1282	3638	3348	1500	8440	1214	1228	8551	2821	1334
**chr17**	1204	3003	2750	1417	8205	1105	1129	7997	2606	1207
**chr18**	1574	3520	3238	1856	8825	1536	1512	8110	2261	1609
**chr19**	725	2197	1693	889	6402	685	696	6179	2001	763
**chr20**	887	2741	2515	1078	6218	859	948	6151	2117	949
**chr21**	868	1596	1419	942	4306	817	767	3704	1112	802
**chr22**	484	1433	1329	575	3278	462	423	2981	1313	432
**chrMT**		3						1		1
**chrUn**	91	168	112	65	414	57	42	1224	241	105
**chrX**	3900	7028	6199	4451	18805	3938	3892	17067	4465	3964
**chrY**	1156	1070	897	1263	3482	1142	1117	3997	896	

**Table A6:** The 10 SSR counts for all the chromosomes of macaca_fascicularis.

	**TAGA**	**TCAT**	**GAAT**	**AGAT**	**AGAA**	**GATA**	**TATC**	**CTTT**	**TCTG**	**TCTA**

chr1	4089	10489	9620	4917	27911	3910	3741	24405	7258	3940
chr2	3811	9063	8322	4380	23717	3508	3804	20824	5701	3930
chr3	3772	8374	7569	4484	23349	3571	3778	20342	5642	3737
chr4	3497	8162	7062	3973	21109	3332	3182	18931	4887	3412
chr5	4086	9340	8308	4837	23873	3988	4018	21130	5093	4176
chr6	3636	8659	7594	4220	24461	3363	3544	20046	5417	3703
chr7	3041	7623	6699	3575	19529	2847	2741	17491	5305	2993
chr8	2999	7070	6210	3575	19030	3064	2904	16280	4465	2798
chr9	2265	5899	5320	2701	15208	2107	2144	13832	4285	2294
chr10	1286	4089	3776	1603	9687	1213	1226	8972	3456	1253
chr11	2578	6338	5856	3378	17447	3038	2410	15251	4211	2579
chr12	2540	6169	5460	2988	15796	2377	2479	14064	3935	2583
chr13	1922	4999	4368	2375	13477	2023	1905	11435	3455	2077
chr14	2322	6079	5608	2886	17473	2419	2303	13383	4176	2394
chr15	2050	5006	4534	2422	12577	1937	1917	11581	3501	2019
chr16	1151	2949	2639	1415	8499	1185	1083	8297	2485	1114
chr17	2075	4781	4013	2364	11759	1987	1976	9999	2721	2044
chr18	1440	3497	3083	1682	8750	1411	1388	7666	2227	1444
chr19	798	1827	1474	1094	8318	1085	724	6486	1849	735
chr20	1240	3269	3110	1565	8415	1482	1169	8299	2648	1203
chrMT					1		2	4		2
chrUn	1289	1968	1125	1590	33522	1489	1394	5179	1208	1250
chrX	3233	7106	5975	3775	19182	3273	3363	16425	4427	3374

**Table A7:** The 10 SSR counts for all the chromosomes of macaca_mulatta.

	**TAGA**	**TCAT**	**GAAT**	**AGAT**	**AGAA**	**GATA**	**TATC**	**CTTT**	**TCTG**	**TCTA**
chr1	3992	10592	9687	4676	26903	3770	3805	23518	7278	4024
chr2	3860	9246	8178	4515	22654	3736	3533	20263	5682	3794
chr3	3620	8366	7519	4354	23341	3440	3673	19989	5648	3624
chr4	3358	7902	6990	3868	20838	3129	3296	17875	4930	3430
chr5	4058	9169	8161	4821	22761	3978	3956	19873	4881	4097
chr6	3620	8568	7478	4191	23428	3337	3512	19346	5376	3699
chr7	2971	7552	6665	3505	18814	2761	2696	16920	5201	2923
chr8	2940	6957	6126	3554	17995	3062	2808	15413	4358	2770
chr9	2288	5829	5257	2687	14584	2135	2150	13343	4223	2277
chr10	1285	3984	3776	1574	9454	1243	1196	8714	3299	1280
chr11	2588	6341	5783	3421	18892	3124	2460	15284	4193	2548
chr12	2075	4997	4462	2421	12608	1944	2010	11210	3107	2084
chr13	2043	4837	4260	2328	12718	1865	2017	11290	3433	1895
chr14	2288	5991	5538	2752	15042	2245	2198	12551	4106	2286
chr15	2029	4956	4484	2386	12241	1926	1886	11039	3447	1970
chr16	1124	2926	2629	1367	8167	1153	1030	7917	2459	1086
chr17	2041	4768	3981	2330	11213	1981	1962	9850	2672	2058
chr18	1423	3365	3006	1701	8557	1358	1395	7483	2181	1429
chr19	758	1802	1486	1002	7446	1063	680	5968	1888	672
chr20	1206	3265	3083	1549	8063	1455	1145	7707	2610	1172
chrMT					1		1	3		1
chrUn	1808	6204	2294	2219	114114	1994	2257	10771	2731	2025
chrX	3279	7094	5925	3792	19977	3297	3351	17104	4393	3322

**Table A8:** The 10 SSR counts for all the chromosomes of nomascus_leucogenys.

	**TAGA**	**TCAT**	**GAAT**	**AGAT**	**AGAA**	**GATA**	**TATC**	**CTTT**	**TCTG**	**TCTA**
chr1a	2300	5504	5024	2684	13392	2136	2182	12578	3503	2318
chr2	3019	7497	6746	3533	17774	2808	2846	16107	4981	3046
chr3	3110	7207	6388	3595	17557	2964	2868	15988	4376	3078
chr4	2758	6588	5938	3300	16612	2681	2866	15108	4578	2947
chr5	3020	6752	6098	3525	16522	2836	2918	14581	4039	3015
chr6	2407	5151	4644	2711	12872	2112	2280	11589	3704	2362
chr7b	2139	5030	4579	2585	12619	2089	2267	11490	3257	2377
chr8	2048	5155	4639	2456	12414	1899	1904	11526	3714	2011
chr9	2621	5658	4892	2972	14597	2463	2328	12155	3187	2485
chr10	1968	4534	4159	2322	11685	1885	1785	10474	3312	1843
chr11	2524	5660	5195	2910	14199	2364	2339	13022	3216	2538
chr12	2062	4839	4587	2423	12463	1968	1929	11405	3221	1991
chr13	1875	4817	4433	2264	12864	1728	1750	10408	3292	1917
chr14	1788	4044	3523	1990	10308	1529	1525	9547	2857	1604
chr15	2087	5438	4916	2506	11784	2007	2110	10651	3413	2181
chr16	2098	4842	4387	2421	11473	2026	1988	10535	2865	2130
chr17	1674	4012	3686	1994	10746	1574	1643	10091	2979	1787
chr18	1864	4479	4025	2132	11676	1682	1787	10588	3117	1808
chr19	1316	3333	3052	1664	8481	1303	1370	7638	2500	1477
chr20	1863	4016	3744	2104	9657	1757	1734	8388	2391	1818
chr21	1793	4101	3730	2099	9865	1665	1670	9225	2457	1735
chr23	679	1545	1426	749	3832	603	590	3441	914	643
chr24	377	1116	1004	454	2333	332	255	2290	1047	289
chr25	685	1380	1224	735	3765	605	642	3164	919	679
chrUn	631	1866	1620	2726	68386	429	426	10441	2745	565
chrX	3394	6496	5803	3957	17985	3487	3754	15256	4125	3700
hr22a	2617	5821	5478	3099	14757	2432	2431	13378	3941	2539

**Table A9:** The 10 SSR counts for all the chromosomes of pan_troglodytes.

	**TAGA**	**TCAT**	**GAAT**	**AGAT**	**AGAA**	**GATA**	**TATC**	**CTTT**	**TCTG**	**TCTA**
chr1	4398	10849	9972	5108	25438	3984	4085	23441	7246	4282
chr2A	2189	4997	4341	2483	12234	1950	1967	11180	3436	1998
chr2B	2747	6208	5655	3149	15379	2522	2558	14008	3938	2672
chr3	4203	9586	8698	4676	23135	3902	3847	20913	5916	4177
chr4	4474	9764	8766	5098	24659	4281	4236	21967	5270	4535
chr5	3880	8633	7663	4436	20696	3606	3500	19659	5246	3921
chr6	3709	8243	7363	4215	20730	3404	3409	19210	5031	3695
chr7	3266	7511	6653	3829	19252	3080	3068	18696	4718	3207
chr8	3107	6972	6350	3661	18035	3073	2870	16424	4307	3005
chr9	2334	5410	4974	2632	13248	2107	2135	12125	3700	2322
chr10	2452	6062	5463	2861	15007	2429	2371	14767	4430	2533
chr11	2565	6304	5849	3062	15090	2457	2464	14012	4440	2643
chr12	2674	6226	5948	3123	15441	2548	2454	15032	3997	2737
chr13	2253	4883	4223	2506	11275	2055	2131	10166	2681	2224
chr14	1731	4208	3706	2063	9677	1646	1634	9501	2754	1692
chr15	1506	3604	3218	1625	8473	1311	1299	7815	2488	1433
chr16	1342	3635	3305	1602	8480	1257	1249	8657	2872	1400
chr17	1275	2989	2791	1510	8195	1147	1038	8007	2670	1163
chr18	1634	3590	3238	1886	8782	1542	1543	8174	2254	1619
chr19	773	2013	1596	834	5858	671	678	6356	1954	784
chr20	933	2817	2615	1074	6116	848	946	6347	2144	981
chr21	758	1504	1305	839	3966	707	690	3366	1028	729
chr22	411	1368	1313	506	3046	390	407	2769	1275	405
chrMT		1					1			
chrUn	909	1470	2395	684	4134	445	536	12435	1409	965
chrX	3338	6609	5848	3859	17083	3427	3384	15111	4071	3438
chrY	535	900	655	717	3547	539	605	3236	931	615

**Table A10:** The 10 SSR counts for all the chromosomes of papio_anubis.

	**TAGA**	**TCAT**	**GAAT**	**AGAT**	**AGAA**	**GATA**	**TATC**	**CTTT**	**TCTG**	**TCTA**
chr1	3872	10425	9563	4687	25568	3783	3780	23446	7273	4049
chr2	3731	9143	8112	4378	22407	3611	3477	20273	5679	3784
chr3	3596	8324	7402	4278	21550	3429	3488	19289	5566	3564
chr4	3250	7818	6868	3775	20282	3047	3143	17631	4811	3273
chr5	3963	9121	8075	4692	22233	3941	3869	19449	4921	4048
chr6	3596	8512	7414	4148	22403	3337	3447	19097	5270	3616
chr7	2967	7460	6613	3570	18373	2792	2646	16586	5115	2868
chr8	2830	6904	6048	3424	17552	2906	2815	15122	4325	2747
chr9	2207	5733	5110	2609	14258	2052	2064	13252	4178	2224
chr10	1272	3937	3693	1547	9226	1239	1149	8721	3237	1256
chr11	2545	6206	5736	3094	15648	2531	2384	14167	4122	2592
chr12	2051	4998	4484	2431	12527	1923	1979	11176	3100	2038
chr13	2434	5844	5083	2762	14789	2150	2272	13142	4182	2263
chr14	2273	6021	5576	2780	14306	2233	2131	12461	4146	2234
chr15	2017	4962	4446	2381	12168	1935	1838	10945	3438	1942
chr16	1097	2903	2613	1243	7918	966	964	7515	2511	1062
chr17	1927	4673	3887	2188	10753	1829	1911	9656	2681	1952
chr18	1446	3454	3069	1680	8644	1392	1411	7546	2157	1411
chr19	702	1814	1428	923	6555	875	650	5522	1825	694
chr20	1152	3236	3041	1390	7817	1238	1131	7721	2591	1152
chrMT										1
chrUn	2366	6016	3788	3392	141376	3667	4305	18080	5860	2499
chrX	3154	6977	5794	3676	18253	3197	3291	15831	4318	3284

**Table A11:** The 10 SSR counts for all the chromosomes of pongo_abelli.

	**TAGA**	**TCAT**	**GAAT**	**AGAT**	**AGAA**	**GATA**	**TATC**	**CTTT**	**TCTG**	**TCTA**
chr1	4779	11685	10749	5677	28928	4554	4343	26348	8064	4696
chr2A	2235	5388	4906	2702	13275	2192	2097	11873	3804	2437
chr2B	2955	6806	6049	3377	16502	2737	2688	14927	4398	2870
chr3	4347	10082	9272	5068	24397	4092	4154	22187	6155	4420
chr4	4801	10531	9292	5616	25309	4611	4660	22367	5751	4874
chr5	4169	9207	8162	4650	22580	3741	3863	19959	5805	4181
chr6	3692	8570	7637	4340	21391	3521	3449	18846	5210	3774
chr7	3261	7578	6898	3856	19078	3076	3274	17161	4852	3396
chr8	3383	7546	6745	3938	18813	3291	3132	16638	4618	3232
chr9	2487	5776	5215	2801	13646	2259	2217	13018	3957	2351
chr10	2597	7135	9532	3061	15947	2470	2459	15064	4703	2716
chr11	2556	6276	5961	3124	14353	2505	2365	12997	4229	2520
chr12	2758	6755	6034	3289	16312	2715	2634	14963	4360	2944
chr13	2404	5229	4512	2754	12205	2267	2279	10795	2874	2421
chr14	1916	4396	3910	2208	10203	1752	1674	9846	2909	1738
chr15	1611	3859	3364	1735	9217	1446	1331	8382	2702	1532
chr16	1399	3763	3438	1624	8575	1326	1303	8706	3165	1420
chr17	1234	3266	3078	1534	8862	1181	1373	8482	2886	1445
chr18	1612	3646	3397	1862	9240	1520	1638	8439	2474	1709
chr19	874	2047	1790	972	6067	744	755	6149	2136	869
chr20	967	3031	2639	1178	6562	907	1034	6248	2295	1089
chr21	946	1694	1490	997	4520	871	747	3925	1125	837
chr22	444	1362	1306	548	3152	416	409	2890	1328	436
chrMT							2			2
chrUn	2257	1972	3392	1107	8138	738	894	13899	3284	2565
chrX	4158	7474	6765	4666	20137	4160	4522	18393	5014	4576

**Table 1:** Genome sequences used to extract 10 SSRs.

**Genome set**	**Number of chromosomes**		**Total Number of Tandem Repeats extracted(≥3)**
**Homo sapiens**	1 to 22, MT, X, Y and Un	(26)	12,83,780
**Callithrix jacchus**	1 to 22, X, Y and Un	(25)	12,21,444
**Chlorocebus sabaeus**	1 to 29, MT, X, Y and Un	(33)	12,48,422
**Gorilla gorilla**	1, 2A, 2B, 3 to 22, MT, X and Un	(26)	12,23,871
**Macaca fascicularis**	1 to 20, MT, X and Un	(23)	
**Macaca mulatta**	1 to 20, MT, X and Un	(23)	13,73,963
**Nomascus leucogenys**	1 to 6, 7b, 8 to 21, 22a, 23 to 25, X and Un	(27)	12,77,214
**Pan troglodytes**	1, 2A, 2B, 3 to 22, MT, X, Y and Un	(27)	13,07,857
**Papio anubis**	1 to 20, MT, X and Un	(23)	13,97,131
**Pongo abelli**	1, 2 A, 2B, 3 to 22, MT, X and Un	(26)	13,88,580
**10**		**259**	

**Table 2:** 10 SSRs successive occurrences for all chromosomes of *homo sapiens and monkeys*.

	**callithrix_jacchus**		**chlorocebus_sabaeus**		**gorilla_gorilla**		**homo_sapiens**		**macaca_fascicularis**	
**SSRs**	**COUNT**	**%**	**COUNT**	**%**	**COUNT**	**%**	**COUNT**	**%**	**COUNT**	**%**
**TAGA**	54,367	4.451	54,232	4.344	55,032	4.497	58,515	4.558	55,120	5.317
**TCAT**	126,046	10.319	129,348	10.361	129,579	10.588	134,401	10.469	132,756	12.805
**GAAT**	125,571	10.281	117,580	9.418	118,095	9.649	121,219	9.442	117,725	11.355
**AGAT**	63,639	5.21	64,076	5.133	64,223	5.248	67,609	5.266	65,799	6.347
**AGAA**	321,541	26.325	337,172	27.008	310,953	25.407	335,774	26.155	383,090	36.952
**GATA**	50,436	4.129	52,584	4.212	51,171	4.181	55,215	4.301	54,609	5.267
**TATC**	49,457	4.049	52,797	4.229	51,226	4.186	55,039	4.287	53,195	5.131
**CTTT**	287,856	23.567	298,314	23.895	299,737	24.491	308,980	24.068	31,032	2.993
**TCTG**	88,988	7.285	87,457	7.005	88,578	7.238	88,741	6.912	88,352	8.522
**TCTA**	53,543	4.384	54,862	4.395	55,277	4.517	58,287	4.54	55,054	5.31
	**macaca_mulatta**		**nomascus_leucogenys**		**pan_troglodytes**		**papio_anubis**		**pongo_abelli**	
**TAGA**	54,654	3.978	54,717	4.284	59,396	4.541	54,448	3.897	63,842	4.598
**TCAT**	1,34,711	9.805	1,26,881	9.934	1,36,356	10.426	1,34,481	9.626	1,45,074	10.448
**GAAT**	1,16,768	8.499	1,14,940	8.999	1,23,903	9.474	1,17,843	8.435	1,35,533	9.761
**AGAT**	65,013	4.732	65,910	5.16	68,038	5.202	65,048	4.656	72,684	5.234
**AGAA**	4,49,811	32.738	3,80,618	29.801	3,36,976	25.766	4,64,606	33.254	3,57,409	25.739
**GATA**	53,996	3.93	51,364	4.022	55,328	4.23	54,073	3.87	59,092	4.256
**TATC**	53,017	3.859	52,187	4.086	55,105	4.213	54,145	3.875	59,296	4.27
**CTTT**	3,03,431	22.084	2,91,064	22.789	3,23,374	24.725	3,06,628	21.947	3,32,502	23.945
**TCTG**	88,096	6.412	84,650	6.628	90,206	6.897	91,306	6.535	98,098	7.065
**TCTA**	54,466	3.964	54,883	4.297	59,175	4.525	54,553	3.905	65,050	4.685

**Table 3:** Example of similarity between child and persons (A and B).

**Child vs**	**Correlation Coefficient**	**Rank Correlation Coefficient**	**Cosine Similarity**
**Person A**	1	1	1
**Person B**	0.22	0.24	0.21

## Appendix A

See Appendix Figs. A1 and A2 and Tables A1–A11 here.
