# Supplementary material for: Data of 10 SSR markers for genomes of homo sapiens and monkeys
Source: Data Brief. 2017 Apr 13;12:287–304. doi: 10.1016/j.dib.2017.04.010 (PMC5407499; doi:10.1016/j.dib.2017.04.010)
Supplement: Supplementary file 1 — Supplementary material [file mmc1.docx]

authors not having the conflict of interest
